# Intravenous Treatment of Acute Articular Rheumatism

**Published:** 1916-04

**Authors:** T. C. Davison

**Affiliations:** Atlanta, Ga.


### INTRAVENOUS TREATMENT OF ACUTE ARTICULAR RHEUMATISM

By Dr. T. C. Davison, Atlanta, Ga.

It is not my intention to read a paper on the treatment of rheumatism or to offer any new treatment, but simply to discuss very briefly a new method of administering old remedies for a very stubborn disease. A few months ago a doctor asked me to tell him what would relieve a case of acute rheumatism? He stated that he had used Phylacogens, also all of the usual remedies, internally, externally and eternally with no results, which had been my experience in some cases.

All of us, I am sure, have had cases of acute rheumatism with delicate stomachs whom we have felt we could relieve, if they only could retain the medicine, but sad to relate they could not. They not only vomit the medicine, but at times their nourishment as well.

The usual internal remedies are very severe upon the strongest stomachs, not to mention the delicate ones.

Therefore, when I heard of a means of relieving acute articular rheumatism intravenously I was very much interested, and was anxious to try it out.

My experience with this method of treating rheumatism has been very limited, and my excuse for bringing the subject before you is to learn the experience and opinion of others, also that if it should appeal to you, you may try it out and let us have further reports later.

I was recently called in consultation to see a case of acute articular rheumatism, an elderly lady with a bad heart, who was subject to repeated attacks and had suffered intensely. The usual remedies had been given and only succeeded in upsetting her stomach and had to resort to opiates to obtain relief and rest. . We used the intravenous treatment and the results were very satisfactory to all parties concerned. Her pain was relieved, she perspired freely, her temperature dropped, and she slept all night without an anodyne. Having omitted the internal medicines she was able to eat and enjoy it next day. The dose was repeated on the third day and in a few days she was up and out.

The treatment should always be preceded by a purging. The treatment consists of:

Sod. Salicylate
Guiacal a a dram i
Glycerine
Aq. Dist. qs., never over 200 c. c.

Take of the above mixture 75 c c, and add normal saline qs. 200 c. c., and give intravenously observing all of the rules of asepsis as in giving any other remedy in this manner.

After giving the treatment wrap the patients up well in hot blankets and let them sweat. There is not necessarily any constitutional reaction.

The patient sweats, kidneys act freely, patient becomes more comfortable and goes off to sleep and has cessation of symptoms in general.

The dose is repeated in 2, 3, 4, or 5 days as indicated.

It is only recommended in acute cases. Although my experience has been very limited, I have had reports on a number of cases in which the results were very gratifying.

914-915 Flat Iron Building, Atlanta.

